# Hydralazine-Induced Isolated Lupus Nephritis

**DOI:** 10.31486/toj.18.0128

**Published:** 2020

**Authors:** Naseer Khan, Ronald Reichel, Ayesha Khurshid, Katie Tso, Joan Broussard, Amrika Dass

**Affiliations:** ^1^ Department of Nephrology, Ochsner Clinic Foundation, Baton Rouge, LA; ^2^ The Institute for Evidence-Based Reform, Baton Rouge, LA; ^3^ Louisiana State University, Baton Rouge, LA; ^4^ Department of Pharmacy, Ochsner Clinic Foundation, Baton Rouge, LA; ^5^ Clinic Operations, Ochsner Clinic Foundation, Baton Rouge, LA

**Keywords:** *Acute kidney injury*, *hydralazine*, *lupus nephritis*, *nephrotic syndrome*, *proteinuria*

## Abstract

**Background:** Hydralazine has been known to cause multiple side effects, both localized and systemic. The literature includes case reports of systemic vasculitis caused by hydralazine.

**Case Report:** A 79-year-old male with stage 3 chronic kidney disease attributable to hypertension and type 2 diabetes was started on hydralazine to control his hypertension. Three weeks after starting hydralazine, the patient developed nephrotic syndrome and acute kidney injury with progressively worsening proteinuria. Pathologic evaluation of the kidney tissue revealed that the patient had lupus nephritis. Immunologic markers confirmed hydralazine-induced lupus nephritis with positive antihistone antibodies. No evidence of systemic vasculitis was found. The patient's hydralazine was stopped, and the patient was treated with immunosuppressive therapy. After 7 months of immunosuppressive therapy, the patient achieved complete remission of lupus nephritis.

**Conclusion:** Isolated renal disease induced by hydralazine as part of drug-induced lupus is uncommon. Our patient developed isolated classic lupus nephritis after hydralazine therapy with no associated systemic vasculitis. Treatment required stopping the hydralazine and initiating systemic immunosuppressive therapy to achieve complete remission.

## INTRODUCTION

Drug-induced lupus erythematosus is a lupus-like syndrome induced by an offending agent or drug. This phenomenon was first described in 1945 in association with sulfadiazine.^1^ Multiple drugs have since been identified to cause drug-induced lupus erythematosus. The drugs most commonly implicated are hydralazine, procainamide, quinidine, isoniazid, diltiazem, minocycline, and tumor necrosis factor-alpha blocking agents.^2^

Hydralazine has been linked to drug-induced lupus erythematosus since the early 1950s.^2-5^ Although rare, most of the systemic syndromes attributed to hydralazine therapy involve multiorgan dysfunction.^6^ Yokogawa and Vivino reviewed 68 cases of systemic vasculitis and lupus-like syndrome attributed to hydralazine and found that the majority of drug-induced lupus erythematosus cases involved systemic vasculitis with a rare renal component (13%).^2^ In the first case series published in 1984, which is the largest to date, 6 cases of drug-induced lupus nephritis were reported.^6^ The majority of the cases involved multiple organ systems with vasculitis and manifestations of pancytopenia, hypocomplementemia, and pulmonary renal syndrome. Overlapping syndromes are present in the majority of renal involvement cases.^7,8^ The Hogan et al review of drug-induced glomerular disease reiterated that hydralazine-induced lupus nephritis is rare.^9^

We present the case of a patient with hydralazine-induced lupus nephritis that resulted in a rapid decline in renal function.

## CASE REPORT

In November 2016, a 79-year-old Caucasian male presented to the nephrology clinic for evaluation of nephrotic syndrome and acute kidney injury with worsening proteinuria and hematuria. The patient's medical history included long-standing essential hypertension, diet-controlled type 2 diabetes, and stage 3 chronic kidney disease for at least 10 years, with stable serum creatinine levels ranging from 1.3 to 1.7 mg/dL. He had no history of proteinuria. Three weeks prior to this visit, the patient's cardiologist initiated hydralazine therapy for management of hypertension. Rheumatology workup for degenerative joint disease in 2015 revealed positive antinuclear antibodies with a ratio of 1:160 without specific antibodies for systemic lupus erythematosus. At that point, rheumatology found no clinical evidence of connective tissue disease.

The patient had had a rheumatologic workup in the past because of symptoms unrelated to his presentation in November 2016 (Table 1). At the patient's presentation in November 2016, urologic workup for hematuria was done (Table 2), and computed tomography urogram showed no blockages or stones. The workup showed nephrotic-range proteinuria and hematuria, along with a sharp rise in serum creatinine, up from a baseline of 1.5 mg/dL (in June 2016) to 1.9 mg/dL (in October 2016) to 2.6 mg/dL (in November 2016). Proteinuria was 5.24 g/24 hours. The patient exhibited features of drug-induced lupus nephritis with positive antihistone antibody and highly positive anti-dsDNA antibody at 1:2560. Complement levels remained within normal limits. Both cytoplasmic antineutrophil cytoplasmic antibody (ANCA) and perinuclear ANCA were negative. Anti–glomerular basement membrane antibodies were negative. Screening with serum protein electrophoresis, cryoglobulins, and anti–smooth muscle antibody tests was negative.

**Table 1.** Patient's Baseline Immunochemistry, 2010-2015

		Laboratory Test Date		
Test	Reference Range	2/10/2010	9/27/2015	12/29/2015
Antinuclear antibody (ANA)	Negative <1:160	Negative		
Antinuclear antibody human epithelial type 2 titer (ANA HEp-2)	Unknown		Positive 1:160	
Anti-Sjögren syndrome related antigen A (anti-SSA), relative units	0.00-19.99		1.62	
Anti-Sjögren syndrome related antigen B (anti-SSB), relative units	0.00-19.99		2.93	
Double-stranded DNA antibody (dsDNA)	Negative 1:10		Negative 1:10	Negative 1:10
Anti-Smith antibody, relative units	0.00-19.99		1.09	
Anti-Smith/ribonucleoprotein antibody, relative units	0.00-19.99		2.17	
Smooth muscle antibody	Negative		Positive 1:40	
Antimitochondrial antibody (indirect fluorescent antibody)	Negative	Negative	Negative 1:40	
Cytoplasmic neutrophilic antibody	<1:20 titer			<1:20
Perinuclear antineutrophil cytoplasmic antibody (P-ANCA)	<1:20 titer			<1:20

**Table 2.** Patient's Immunochemistry at Presentation and Posttreatment, 2016-2017

		Laboratory Test Date						
Test	Reference Range	Presentation and Admission			Discharge	Posttreatment		
		11/17/16	11/22/16	11/22/16	11/24/16	12/6/16	1/25/17	3/21/17
Double-stranded DNA antibody (dsDNA)	Negative 1:10	Positive 1:2560			Positive 1:2560		Positive 1:20	Negative 1:10
Anti-Smith antibody, relative units	0.00-19.99	5.04						
Antihistone antibody, units	0.0-0.9			3.5				
Cytoplasmic neutrophilic antibody	<1:20 titer		<1:20					
Perinuclear antineutrophil cytoplasmic antibody (P-ANCA)	<1:20 titer		<1:20					
Proteinase 3 antineutrophil cytoplasmic antibody (PR3-ANCA)	Negative <0.4		<0.2					
Complement 3 (C3), mg/dL	50-180	71		65		53	120	144
Complement 4 (C4), mg/dL	11-44	13		13		11	34	40
Anti-glomerular basement membrane antibody, units	<1.0		<0.2			<0.2		
Computed tomography urology		Negative						

**Figure 1.**
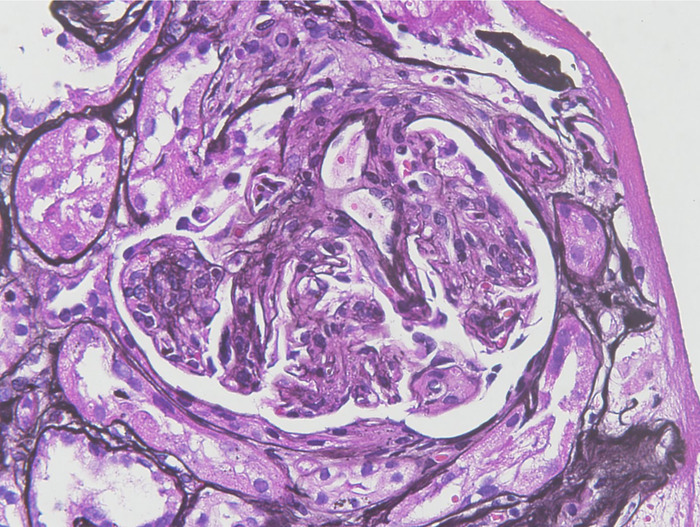
**Light microscopy shows diffuse proliferative glomerulonephritis (magnification ×40).**

**Figure 2.**
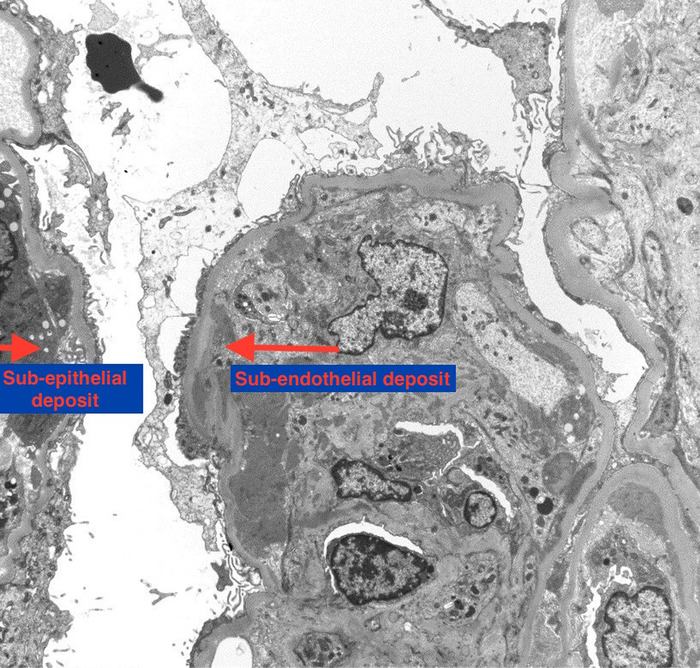
**Electron microscopy shows immunologic damage to kidney with antibody deposits: thickened glomerular basement membrane; occasional subendothelial, rare subepithelial and scattered mesangial deposits; and rare reticular aggregates (magnification ×2800).**

**Figure 3.**
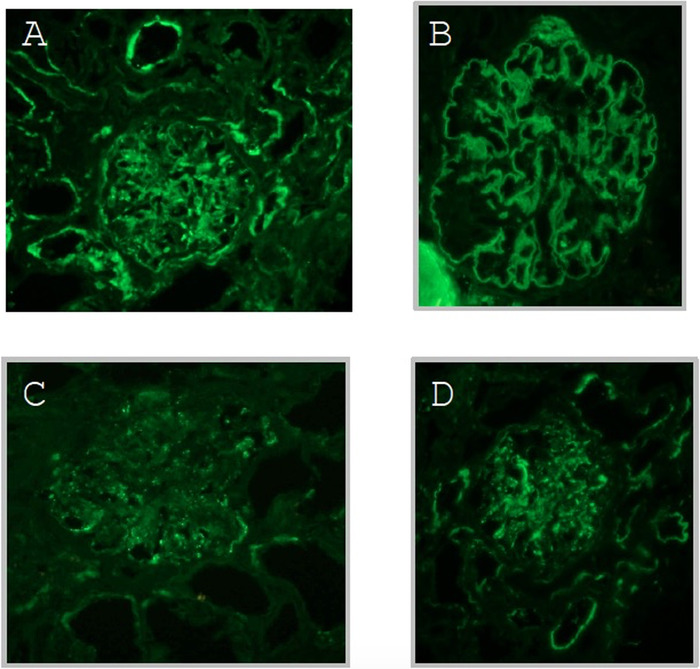
**Demonstration of (A) immunoglobulin (Ig) G, (B) IgA, (C) complement component 1q (C1q), and (D) complement 3 component (C3) antibodies on immunofluorescence is consistent with the clinical picture of lupus nephritis. Images show codominant IgG and IgA (1-2+) diffuse segmental to global mesangial and capillary loop staining in a full house pattern and diffuse granular basement membrane staining for IgG, IgA, C1q, and C3.**

The patient was advised to discontinue hydralazine; however, his renal function did not improve upon drug discontinuation. His serum creatinine peaked at 5.2 mg/dL with a glomerular filtration rate <10 mL/min. Diagnostic kidney biopsy revealed diffuse proliferative glomerulonephritis (Figures 1 and 2). Immunofluorescence was consistent, with multiple immune deposits as seen in lupus nephritis (Figure 3). Based on these findings, the patient was diagnosed with hydralazine-induced lupus nephritis.

Because renal function did not improve upon discontinuation of hydralazine, the patient was started on combination immunosuppressive therapy that consisted of a daily dose of intravenous methylprednisolone (Solu-Medrol) 1,000 mg daily for 3 days and then continued on oral mycophenolate mofetil 500 mg twice daily and oral prednisone 60 mg daily for the first month (November 2016). His dosage of oral mycophenolate mofetil was increased to 1,000 mg twice daily the following month. Beginning in January 2017, prednisone was tapered to 10 mg daily. In February, mycophenolate mofetil therapy was discontinued, at which time the patient developed pneumonia and shingles. The patient continued on a tapered dose of prednisone for 3 months, after which he was continued on prednisone 10 mg for an additional 4 months (Table 3).

**Table 3.** Prescribed Medications With Doses

Date	Medications
October 2016	Hydralazine 25 mg twice daily was initiated.
November 2016	Hydralazine stopped. Administered intravenous methylprednisolone (Solu-Medrol) 1,000 mg pulse dose daily for 3 days and then began prednisone 60 mg daily and mycophenolate mofetil 500 mg twice daily.
December 2016	Prednisone taper started. Initial prednisone dose was 60 mg daily for 1 week. Dose was decreased by 10 mg each subsequent week. Mycophenolate mofetil was increased to 1,000 mg twice daily.
January 2017	Prednisone tapered to 10 mg daily.
February 2017	Patient developed pneumonia and shingles. Mycophenolate mofetil therapy discontinued. Prednisone 10 mg daily dose continued.
May 2017	Prednisone tapered to discontinuation.

The patient responded to combination therapy with steroids and mycophenolate mofetil. His renal function progressively improved, and his dsDNA antibody levels decreased. The patient achieved complete remission of lupus nephritis with resolution of proteinuria, and his kidney function returned to baseline after 3 months. After 7 months of immunosuppressive therapy, all immunologic markers returned to the patient's baseline levels (Table 2).

In this case, the only clinical manifestation of drug-induced lupus erythematosus was lupus nephritis that caused a decline in kidney function and nephrotic-range proteinuria. Treatment required aggressive immunosuppressive therapy and the cessation of hydralazine.

## DISCUSSION

Hydralazine is a commonly prescribed hypertensive agent that has a wide variety of clinical uses, from treatment of essential hypertension to treatment for congestive heart failure. Hydralazine can be prescribed alone or in combination with other medications. Systemic side effects of hydralazine are rare. Some of the adverse effects related to hydralazine include reflex tachycardia, hemolytic anemia, vasculitis, glomerulonephritis, and lupus-like syndrome.^10,11^ Although our patient had a history of hypertension and type 2 diabetes, he had stable kidney function with no heavy proteinuria for many years prior to the introduction of hydralazine. At the time of the patient's presentation, the differential diagnosis included diabetic nephropathy, hypertensive nephropathy, and drug-induced glomerulonephritis. Patients with type 2 diabetes and hypertension can develop nephrotic-range proteinuria due to diabetic nephropathy and hypertensive nephropathy. Although our patient had both hypertension and type 2 diabetes, we suspected the possibility of glomerulonephritis given the rapid onset of nephrotic-range proteinuria and the rapid decline of kidney function. Further investigation with immunologic markers revealed the presence of antihistone antibodies that fulfilled the criteria for drug-induced lupus erythematosus. Both clinically and on immunologic workup, the only manifestation of systemic lupus erythematosus was glomerulonephritis. Biopsy confirmed lupus nephritis.

Our case fulfilled the clinical and immunologic criteria for lupus nephritis (ie, hydralazine-induced systemic lupus erythematosus confined to the kidney). The final diagnosis of lupus nephritis without evidence of systemic vasculitis is in sharp contrast to what was described in the literature with cases that involved overlapping vasculitic diseases. Some cases of systemic lupus erythematosus were not confined to renal involvement only; some cases had multiorgan involvement, including lung and skin.^6^

The collaborative treatment team included cardiology, nephrology, and rheumatology. Because of the patient's advanced age and multiple comorbidities, we chose mycophenolate mofetil for immunosuppression instead of cyclophosphamide in addition to the steroids typically used to treat lupus nephritis. The patient achieved complete remission of lupus nephritis after 7 months of combination immunosuppressant therapy.

Multiple drugs, including hydralazine, have been implicated in inducing an autoimmune response, resulting in drug-induced glomerulonephritis.^9^ Most of these drug-induced glomerulonephritis cases involved systemic vasculitis syndrome. ANCA testing is used to diagnose types of autoimmune vasculitis.^12^ The patients described in the 1984 study were not screened for ANCA; consequently, they could have had overlapping syndromes that were not diagnosable at the time because of limitations in available testing.^6^ In our case, we were able to successfully exclude ANCA-associated vasculitis.

Positive antihistone antibody and ANA HEp-2 titer are typically seen in hydralazine-induced lupus.

Based on the available literature, risk factors for hydralazine-induced lupus include old age, being Caucasian, slow acetylator phenotype, and human leukocyte antigen – DR isotype (HLA-DR4) genotype.^2,5^ We accept the limitation of our workup for hydralazine-induced lupus nephritis, as we did not have slow acetylator or HLA-DR4 status for our patient.

## CONCLUSION

Reports of hydralazine-induced isolated lupus nephritis without evidence of any other systemic vasculitis are rare in the literature. Our case of isolated classic lupus nephritis with no associated systemic vasculitis induced by hydralazine required the cessation of hydralazine and systemic immunosuppressive therapy. Strong clinical suspicion, prompt immunologic evaluation, and diagnostic renal pathology were key to proper management and successful treatment.

## Notes

This article meets the Accreditation Council for Graduate Medical Education and the American Board of Medical Specialties Maintenance of Certification competencies for Patient Care and Medical Knowledge.

## References

[1] Hoffman BJ Sensitivity to sulfadiazine resembling acute disseminated lupus erythematosus. Arch Derm Syphilol. 1945;51(3):190. doi: 10.1001/archderm.1945.01510210032007.

[2] Yokogawa N, Vivino FB Hydralazine-induced autoimmune disease: comparison to idiopathic lupus and ANCA-positive vasculitis. Mod Rheumatol. 2009;19(3):338-347. doi: 10.1007/s10165-009-0168-y. 19424772

[3] Cameron HA, Ramsay LE. The lupus syndrome induced by hydralazine: a common complication with low dose treatment. Br Med J (Clin Res Ed). 1984 Aug 18;289(6442):410-412. doi: 10.1136/bmj.289.6442.410. PMC1442447 6432120

[4] Marzano AV, Lazzari R, Polloni I, Crosti C, Fabbri P, Cugno M Drug-induced subacute cutaneous lupus erythematosus: evidence for differences from its idiopathic counterpart. Br J Dermatol. 2011 8;165(2):335-341. doi: 10.1111/j.1365-2133.2011.10397.x. 21564069

[5] Shapiro KS, Pinn VW, Harrington JT, Levey AS Immune complex glomerulonephritis in hydralazine-induced SLE. Am J Kidney Dis. 1984 1;3(4):270-274. doi: 10.1016/s0272-6386(84)80044-x. 6229178

[6] Ihle BU, Whitworth JA, Dowling JP, Kincaid-Smith P Hydralazine and lupus nephritis. Clin Nephrol. 1984 11;22(5):230-238. 6240359

[7] Kalra A, Yokogawa N, Raja H, Hydralazine-induced pulmonary-renal syndrome: a case report. Am J Ther. 2012 7;19(4):e136-e138. doi: 10.1097/MJT.0b013e3181ed838c. 20724911

[8] Keasberry J, Frazier J, Isbel NM, Van Eps CL, Oliver K, Mudge DW Hydralazine-induced anti-neutrophil cytoplasmic antibody-positive renal vasculitis presenting with a vasculitic syndrome, acute nephritis and a puzzling skin rash: a case report. J Med Case Rep. 2013 Jan 14;7:20. doi: 10.1186/1752-1947-7-20. 23316942 PMC3565908

[9] Hogan JJ, Markowitz GS, Radhakrishnan J Drug-induced glomerular disease: immune-mediated injury. Clin J Am Soc Nephrol. 2015 Jul 7;10(7):1300-1310. doi: 10.2215/CJN.01910215. 26092827 PMC4491282

[10] Hydralazine side effects. Drugs.com. www.drugs.com/sfx/hydralazine-side-effects.html. Updated January 23, 2019. Accessed December 13, 2019.

[11] Label: Hydralazine - hydralazine hydrochloride tablet. U.S. National Library of Medicine DailyMed. dailymed.nlm.nih.gov/dailymed/drugInfo.cfm?setid=1fd8cf42-66ae-4af5-a5ae-7c9679a0e532. Updated February 15, 2017. Accessed December 13, 2019.

[12] Savige J, Pollock W, Trevisin M What do antineutrophil cytoplasmic antibodies (ANCA) tell us? Best Pract Res Clin Rheumatol. 2005 4;19(2):263-276. doi: 10.1016/j.berh.2004.10.003. 15857795

